# Enavatuzumab, a Humanized Anti-TWEAK Receptor Monoclonal Antibody, Exerts Antitumor Activity through Attracting and Activating Innate Immune Effector Cells

**DOI:** 10.1155/2017/5737159

**Published:** 2017-09-17

**Authors:** Shiming Ye, Melvin I. Fox, Nicole A. Belmar, Mien Sho, Debra T. Chao, Donghee Choi, Yuni Fang, Vivian Zhao, Stephen F. Keller, Gary C. Starling, Patricia A. Culp

**Affiliations:** ^1^AbbVie Biotherapeutics Inc., Redwood City, CA 94063, USA; ^2^Merck, Palo Alto, CA 94304, USA; ^3^Alector LLC, South San Francisco, CA 94080, USA

## Abstract

Enavatuzumab is a humanized IgG1 anti-TWEAK receptor monoclonal antibody that was evaluated in a phase I clinical study for the treatment of solid malignancies. The current study was to determine whether and how myeloid effector cells were involved in postulated mechanisms for its potent antitumor activity in xenograft models. The initial evidence for a role of effector cells was obtained in a subset of tumor xenograft mouse models whose response to enavatuzumab relied on the binding of Fc of the antibody to Fc*γ* receptor. The involvement of effector cells was further confirmed by immunohistochemistry, which revealed strong infiltration of CD45^+^ effector cells into tumor xenografts in responding models, but minimal infiltration in nonresponders. Consistent with the xenograft studies, human effector cells preferentially migrated toward *in vivo*-responsive tumor cells treated by enavatuzumab *in vitro*, with the majority of migratory cells being monocytes. Conditioned media from enavatuzumab-treated tumor cells contained elevated levels of chemokines, which might be responsible for enavatuzumab-triggered effector cell migration. These preclinical studies demonstrate that enavatuzumab can exert its potent antitumor activity by actively recruiting and activating myeloid effectors to kill tumor cells. Enavatuzumab-induced chemokines warrant further evaluation in clinical studies as potential biomarkers for such activity.

## 1. Introduction

Functional antibodies targeting cell surface receptors with the ability to induce signaling represent a relatively new class of therapeutic antibodies. Members of the TNF receptor super family (TNFRSF) are attractive targets for developing functional antibodies since stimulating this class of receptors potently regulates a wide variety of biological functions. Several antibodies targeting the members of TNFRSF, such as anti-TRAILR, anti-OX40, anti-CD40, and anti-4-1BB, have been developed and evaluated in preclinical studies or in clinical trials [1]. Enavatuzumab (also called PDL192) is a humanized IgG1 monoclonal antibody targeting the receptor of TNF-like weak inducer of apoptosis (TWEAK), one of the TNFRSF members, also known as Fn14 or TNFRSF12A [2, 3]. TWEAK is the natural ligand of the TWEAK receptor (TweakR), which stimulates multiple cellular responses, including proliferation, differentiation, apoptosis, and migration, as well as wound repair and inflammation [4, 5].

Although TWEAK has pleiotropic functions, it was initially identified as a weak inducer of apoptosis [6]. Additional studies further indicated that TWEAK can induce multiple cancer cell lines to undergo caspase-dependent apoptosis, and cell death can be further enhanced when combined with TNF*α* or IFN*γ* treatment [7–9]. Since TweakR is overexpressed in multiple tumors, such as breast cancer, lung cancer, ovarian cancer, glioma, and endometrial cancer [10–15], several functional anti-TweakR antibodies have been investigated for treating cancers [16]. Due to the relatively low expression of TweakR in normal tissues, an immunotoxin-conjugated TweakR antibody has been tested in preclinical cancer models [17, 18]. We also reported previously that the antitumor activity of enavatuzumab has been attributed to three distinct mechanisms of action: (1) direct killing of tumor cells by inducing caspase-3/7 activation, (2) growth inhibition of tumor cell lines through p21-mediated cell cycle arrest, and (3) via antibody dependent cell-mediated cytotoxicity (ADCC) [2, 19].

Depletion of target cells through ADCC has been implicated as a major mechanism for therapeutic antibodies, including rituximab, alemtuzumab, and trastuzumab in treating both hematologic malignancies and solid tumors [20]. In addition to this conventional role in mediating ADCC, the interaction of Fc and the Fc*γ* receptor (Fc*γ*R) also provides a means to crosslink the antibodies, which for agonist antibodies enhances their signaling potential upon binding to the target antigen, as was recently confirmed for anti-DR5 antibodies [21]. The availability of Fc receptor-positive cells is therefore critical for antibodies to function through ADCC as well as through targets. Results from clinical studies in breast cancer patients indicate that increased numbers of lymphocytes within the tumor correlates with improved response to trastuzumab [22]. Thus, the presence of effector cells within the tumor microenvironment may be required for antibodies to best achieve their antitumor activities. Although ADCC function has been extensively studied preclinically for many therapeutic antibodies, very few studies have focused on whether therapeutic antibodies can actively recruit Fc*γ*R-positive cells into the tumor microenvironment, which may enhance ADCC or agonistic activity of therapeutic mAb *in vivo*.

As previously described, enavatuzumab induces tumor growth inhibition through direct TweakR signaling and ADCC. However, this may be an oversimplification of the mechanisms of this antibody given the pleiotropic nature of TweakR-mediated signaling. In the current study, we focused on the interaction of enavatuzumab with tumor cells and immune effector cells. We found that enavatuzumab treatment results in activation of immune effector cells and infiltration of immune cells into the tumors in mice bearing xenograft tumors sensitive to the antibody. We also showed that enavatuzumab stimulates migration of human immune cells *in vitro* toward tumor cells sensitive to enavatuzumab and that MCP-1 is a key driver of this migration. MCP-1 was also found to be increased in the serum of mice and in human patients after enavatuzumab treatment, suggesting that the preclinical findings may translate into the clinical setting.

## 2. Methods

### 2.1. Cell Lines and Therapeutic Antibodies

Tumor cell lines H520, A375, HCT116, and DLD-1 cells were obtained from ATCC, while SN12C was purchased from NCI. H520 lung cancer cells, SN12C renal cancer cells, and HCT116 and DLD-1 colorectal cancer cells were maintained in RPMI, and A375 melanoma cells were maintained in DMEM. H520 cells were transfected with a TweakR expression construct to generate H520-TweakR cell line. All cells were maintained and assays were done in the appropriate growth media containing fetal bovine serum (10%), unless otherwise indicated. All cell culture media and serum were purchased from Hyclone (Thermo Fisher Scientific).

Enavatuzumab and the human IgG1 isotype control (MSL109) have been described previously [2]. The enavatuzumab Fc mutant 1 is on a human IgG1 backbone that contains the L234A/L235A mutations in the Fc region (huIgG1-LALA), while the enavatuzumab Fc mutant 2 variant is a human IgG2 isotype containing the V234A/G236A mutations (hIgG2-VAGA).

### 2.2. Animal Models

Tumor cells were inoculated subcutaneously into the right flank of 6-week old severe combined immunodeficient (SCID) mice (IcrTac:ICR-Prkdc^<scid>^, Taconic, Germantown, NY) at 1 × 10^7^ cells per mouse. Animals were randomized into groups when the mean tumor volume reached 110–160 mm^3^. Antibodies were administered intraperitoneally at 10 mg/kg, unless otherwise indicated.

For efficacy studies, tumor volumes (L × W × H/2) were generally measured on each dosing day; the group means ± SEM is displayed. Groups were removed from the study when at least one tumor in the group reached the allowable limit (1500 mm^3^). The statistical significance of the differences between groups was determined by *t*-test using SAS statistical software (version 9). Mean tumor volumes between groups were considered significantly different if *p* < 0.05.

For tumor samples collected for immunohistochemistry, animals were administered antibody on days 0 and 2 or 3, and tumors were harvested on day 4.

For cytokine measurements, A375 tumor-bearing mice were given a single dose of antibody, and blood samples were taken up to 14 days after antibody dose. Cytokine levels were measured in serum by Luminex® (Millipore, Billerica, MA), according to the manufacturer's instructions.

All animal work was carried out under NIH guidelines “Guide for the Care and Use of Laboratory Animals” using AbbVie Biotherapeutics IACUC approved protocols.

### 2.3. Phenotyping of Mouse Splenocytes

SN12C or HCT116 tumor-bearing mice were given 7 or 9 doses, respectively, of enavatuzumab or a control antibody (10 mg/kg three times per week). Three days after the last antibody dose, spleens were harvested from 5–7 mice in each group, and isolated splenocytes were stained with conjugated staining antibodies from BD Bioscience (San Jose, CA): CD45-FITC, CD11b-APC-Cy7, DX5-PE, and biotinylated CD27. FACS data were collected by FACSCanto™ (BD Biosciences, San Jose, CA) and analyzed with Flowjo (Tree Star, Ashland, OR).

### 2.4. Antibody-Dependent Cell-Mediated Cytotoxicity (ADCC) Assay

The ADCC activity of enavatuzumab wild-type or mutant antibodies was measured by Cr-51 release as described previously [2] using human peripheral blood mononuclear cells (PBMCs) as effectors and TweakR-positive tumor cells as targets. In brief, target cells were labeled with 50 *μ*Ci of Cr-51 (Perkin Elmer, Waltham, MA) per 1 × 10^6^ cells for 1 hour (hr) at 37°C. Labeled target cells were mixed and incubated with serially diluted antibody for 30 min at 4°C. PBMCs were prepared from fresh whole blood using a Ficoll-Paque Plus gradient (GE Healthcare Biosciences, Pittsburgh, PA). PBMCs were then added to the opsonized cells at a E : T ratio at 40 : 1 and incubated for 4 hrs at 37°C in a CO_2_ incubator. Antibody-independent cell-mediated cytotoxicity (AICC) was measured by incubating effector and target cells in the absence of antibody. Maximum release (MR) was measured by adding 2% Triton X-100 to target cells. Spontaneous release (SR) was measured by incubating target cells in the absence of antibody. After 4 hrs, the plates were gently centrifuged and Cr-51 release was measured by counting 100 *μ*l of cell-free supernatant in a Wizard 1470 gamma counter (Perkin Elmer). The percent cytotoxicity was calculated as [(Sample − SR)/(MR − SR)] × 100.

### 2.5. *In Vitro* Coculture Assay

PBMCs from healthy human donors were added to 24-well plates, either alone or into wells that contained SN12C cells that had been plated 24 hrs previously. The cultures were incubated with enavatuzumab or a control antibody (10 *μ*g/mL) for 24 hrs, after which the immune cells were removed to measure activation markers by flow cytometry. Immune cells were stained for monocytes and nature killer (NK) cells with fluorochrome-conjugated antibodies purchased from BD Biosciences: CD3-FITC, CD54-PE, CD16-PerCP Cy5.5, CD14-PE Cy7, CD56-APC, and CD69-APC Cy7. In some experiments, tumor cell cytotoxicity was assessed after 24 hr culture by measuring the level of cytokeratin18 in the supernatant by M65® ELISA (Peviva, Bromma, Sweden), according to the manufacturer's instructions.

### 2.6. *In Vitro* Migration Assay

A total of 6 × 10^4^ tumor cells were plated into the bottom well of 24-well Transwell® plates (Corning Inc., Corning, NY) and incubated with antibodies (10 *μ*g/mL). Twenty-four hours later, 2 × 10^5^ PBMCs from healthy donors were added to the top well (5 *μ*m) of the Transwell plate and incubated for additional 4 hrs. Wells containing no tumor cells in the bottom chamber were used to quantify spontaneous migration. In some experiments, anti-human MCP-1 or anti-human IL-8 (R&D Systems, Minneapolis, MN) was added 30 min before the addition of PBMC. The total number of immune cells that had migrated into the bottom well was quantified by FACS using polystyrene beads (Polysciences, Warrington PA). Specific migration was calculated by 100 × ((number of cells that migrated toward tumor cells − number of cells that migrated spontaneously)/number of PBMCs seeded). In some experiments, the supernatants from antibody-treated tumor cells were measured for cytokine production by Luminex, and immune cells that had migrated were phenotyped by collecting migrated cells and staining them with antibodies from BD Biosciences: CD3-FITC, CD56/CD16-PE, CD4-PerCP Cy5.5, CD14-PE Cy7, CD11c-APC, and CD20-APC Cy7. Analysis was performed on a FACSCanto flow cytometer.

### 2.7. Immunohistochemistry Staining

Tumor xenografts were harvested, fixed in 10% buffered formalin, and embedded in paraffin. Five-micrometer sections were deparaffinized, and antigen retrieval was performed in BORG solution (Biocare Medical, Concord, CA), followed by blocking with Background Sniper (Biocare Medical). Slides were incubated with anti-mouse CD45 antibody (rat IgG2b) or a control rat IgG2b antibody (BD Bioscience) and detected using the Rat on Mouse HRP detection system (Biocare Medical). Slides were then incubated with diaminobenzidine for 5 minutes and were counterstained with hematoxylin. Mouse effector cells were identified by CD45-positive staining.

### 2.8. Cytokine Measurements in Human Serum Samples

Serum samples were collected from patients in the enavatuzumab phase 1 study according to the study protocol. A 12-plex Luminex assay from Millipore was validated to measure cytokine/chemokine/growth factor levels in human serum. The assay kit contains capture antibodies for each analyte covalently bound to distinct color-coded microsphere subsets distinguished by differing dye ratios. Calibrators, controls, and study samples were incubated with microspheres in the wells of 96-well plates. The assay signal for each individual analyte was determined by measuring orange fluorescence produced by a complex of biotinylated analyte-specific antibodies and streptavidin-phycoerythrin as fluorescence intensity (FI) using a Luminex XMAP instrument. The concentration of each analyte was determined by using the FI value to extrapolate against the calibration curve generated from the regression of the FI values of the calibrators and their corresponding nominal concentrations.

### 2.9. Statistical Analysis

Data were analyzed using SAS statistical software (version 9) and GraphPad Prism software, version 4.03. They were subjected to one-way (treatment) ANOVA. When ANOVA revealed a significant effect of treatment, differences between treatments were tested using Duncan's multiple range tests.

## 3. Results

### 3.1. Enavatuzumab Exerted Potent ADCC on TweakR-Positive Tumor Cells through Activating Immune Effector Cells *In Vitro*

**Figure 1:**
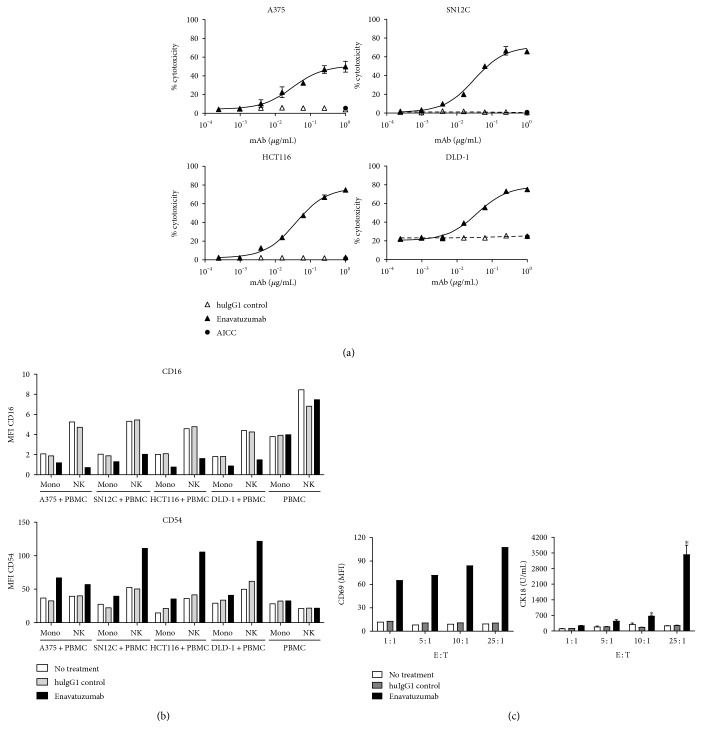
Enavatuzumab induces effector cell activation and tumor cell killing *in vitro*. (a) Human PBMCs were coincubated with Cr-51-labeled tumor target cells with E : T at 40 : 1 for 4 hr in the presence of enavatuzumab. Tumor cell cytotoxicity was calculated based on Cr-51 released into culture supernatant. Antibody-independent cell cytotoxicity (AICC) was also calculated. Experiments were performed with PBMCs from 4 donors. Representative data collected from one donor are shown. (b) Human PBMCs, cultured either alone or with indicated tumor cells at a 10 : 1 ratio, were treated with enavatuzumab or a control antibody. 24 hrs later, CD54 and CD16 levels were assessed on both monocytes (Mono) and NK cells by flow cytometry. Experiments were performed with PBMCs from 8 donors. Representative data collected from one donor were shown. (c) PBMCs were cultured with SN12C cells at the indicated ratios for 24 hr in the presence of enavatuzumab or a control antibody, after which CD69 was assessed on NK cells by flow cytometry (left) or cytokeratin18 levels were quantified in cell supernatants as a measure of tumor cell cytotoxicity (right). Enavatuzumab treatment increased CD69 levels at all E : T ratios tested (representative data from 4 donors) but significantly stimulated cytotoxicity only at 10 : 1 and 25 : 1 ratios (*n* = 4, ^∗^*p* < 0.05).

Enavatuzumab is a humanized IgG1 antibody, and Fc-mediated effector cell killing has been proposed as one of the mechanisms driving its antitumor activity. We previously reported that enavatuzumab ADCC on cells transfected with TweakR [2]. To further confirm the ability of enavatuzumab to induce ADCC *in vitro*, ADCC was evaluated on multiple endogenous TweakR-expressing tumor cell lines as targets [2]. Enavatuzumab showed potent tumor cell killing on all TweakR-positive tumor cells tested, including the renal carcinoma cell line SN12C, the melanoma cell line A375, and the colorectal cancer cell lines HCT116 and DLD-1 (Figure 1(a)).

ADCC is generally thought to be mediated by the activation of immune effector cells. To explore this further, immune cell activation by enavatuzumab was assessed in *in vitro* cocultures of human PBMCs and tumor cells. When PBMCs were cultured, enavatuzumab treatment did not alter the expression of CD54 and CD16 on monocytes or NK cells. In contrast, enavatuzumab treatment of cocultures of PBMCs with any of the four TweakR-expressing tumor cell lines resulted in activation of both monocytes and NK cells, as defined by upregulation of CD54 and downregulation of CD16 on both cell types (Figure 1(b)). To determine whether effector cell activation is sufficient to mediate tumor cell killing, we compared enavatuzumab-induced effector cell activation and tumor cell killing across a range of effector : target ratios (E : T). In cocultures of PBMCs and SN12C cells, NK activation, indicated by CD69 upregulation, was observed over a range of E : T, from 1 : 1 to 25 : 1 (Figure 1(c)). In contrast, enavatuzumab induced cytotoxicity of SN12C cells, indicated by CK18 production, started at E : T of 10 : 1, with marked cytotoxicity observed only at the highest E : T tested (25 : 1, Figure 1(c)). At high E : T, enavatuzumab stimulated cytotoxicity of all TweakR-expressing cells equivalently *in vitro* (Figure 1(a)). These data suggest that the quantity of effector cells is also essential for mediating tumor cell killing in addition to effector cell activation.

### 3.2. Enavatuzumab Induced Diverse Responses on TweakR-Positive Xenograft Tumors by Differentially Activating Immune Effector Cells *In Vivo*

**Figure 2:**
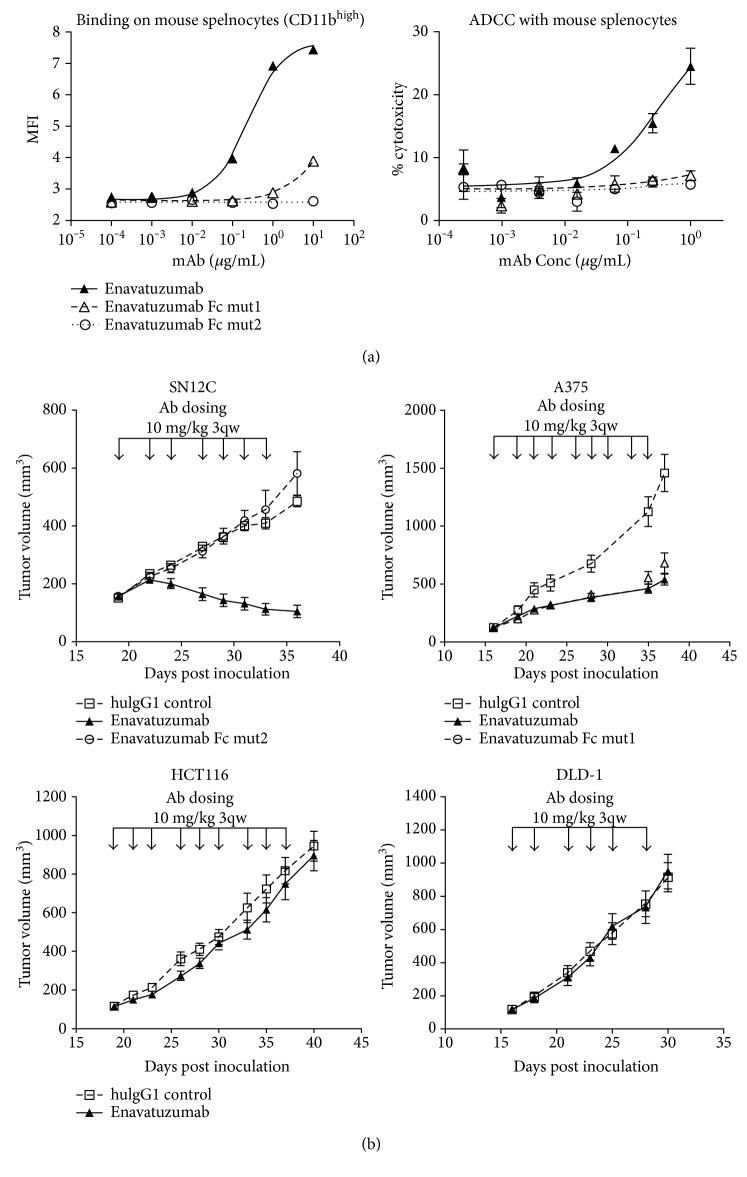
Enavatuzumab showed diverse antitumor activities on different xenograft tumors. (a) Enavatuzumab or two variants of enavatuzumab containing mutations in the Fc region were incubated with mouse splenocytes. Binding to CD11b^high^ cells was measured by FACS. ADCC activities of enavatuzumab and its Fc mutant variants were evaluated by Cr-51 release with H520-TweakR cells as targets and mouse splenocytes as effectors at a ratio of 1 : 40. (b) Established SN12C tumors were treated with enavatuzumab, a variant of enavatuzumab with no Fc*γ*R binding, or a control antibody (10 mg/kg) three times a week for a total of seven doses, with dosing days indicated by the arrows above the graph. Dosing groups contained 10 animals each. Treatment with enavatuzumab, but not the Fc mutant, resulted in significant tumor growth inhibition on days 24–36 (*p* < 0.05). A375 tumors were similarly administered with nine doses of enavatuzumab, an Fc mutant variant, or a control antibody. Dosing groups contained 8 animals each, and significant growth inhibition was observed with both enavatuzumab and the Fc mutant on days 21–37 (*p* < 0.05). HCT116 and DLD-1 xenograft tumors were treated with enavatuzumab or a control antibody for nine or six doses (*n* = 8 or 10). Enavatuzumab treatment resulted in no tumor growth inhibition in either models.

We next attempted to translate the ability of enavatuzumab to kill a range of tumor cell lines via ADCC *in vitro* into antitumor activity *in vivo*. Enavatuzumab has previously been shown to exhibit potent antitumor activity on human xenograft tumors implanted into ICR-SCID mice. ICR-SCID mice maintain a largely intact innate immune system and produce effector cells that express multiple Fc*γ* receptors. Enavatuzumab has been shown to induce ADCC *in vitro* using ICR-SCID mouse splenocytes as effector cells [2], suggesting that the human IgG1 Fc is able to bind effectively to mouse Fc*γ* receptors [23]. This binding was confirmed in flow cytometry assays measuring enavatuzumab binding on mouse CD11b^high^ splenocytes (Figure 2(a)). This interaction is likely through Fc-Fc*γ*R, as TweakR expression has not been detected on lymphoid cell types, regardless of their activation state [2, 24]. Moreover, enavatuzumab does not bind the mouse ortholog of human TweakR (data not shown). When tested on a range of xenograft tumor models, however, not all TweakR-positive tumors showed similar *in vivo* responses to enavatuzumab treatment. Some TweakR-expressing tumor cell lines, such as HCT116 and DLD-1, were not sensitive to enavatuzumab treatment *in vivo* (Figure 2(b)), though both cell lines were effectively killed via ADCC *in vitro* with enavatuzumab. Other TweakR-expressing cells, such as SN12C and A375, were sensitive to enavatuzumab treatment both *in vivo* and *in vitro*. However, the *in vivo* responses to enavatuzumab treatment in these cell lines appeared to rely on different mechanisms of action. In the SN12C model, an enavatuzumab Fc mutant variant, Fc mutant 2 (hIgG2-VAGA), with no binding to Fc*γ*R and no ADCC capability (Figure 2(a)), was unable to inhibit the growth of tumors, suggesting a critical role for Fc-Fc*γ*R interaction and/or ADCC in this model (Figure 2(b)). In contrast, a different Fc mutant variant, Fc mutant 1 (hIgG1-LALA), which was unable to induce ADCC, but retained some binding to mouse Fc*γ*R-expressing cells (Figure 2(a)), inhibited the growth of A375 tumors to a similar extent as wild-type enavatuzumab (Figure 2(b)), suggesting that cell death signaling through TweakR, and not ADCC, is critical for the antitumor activity of enavatuzumab in this model.

**Figure 3:**
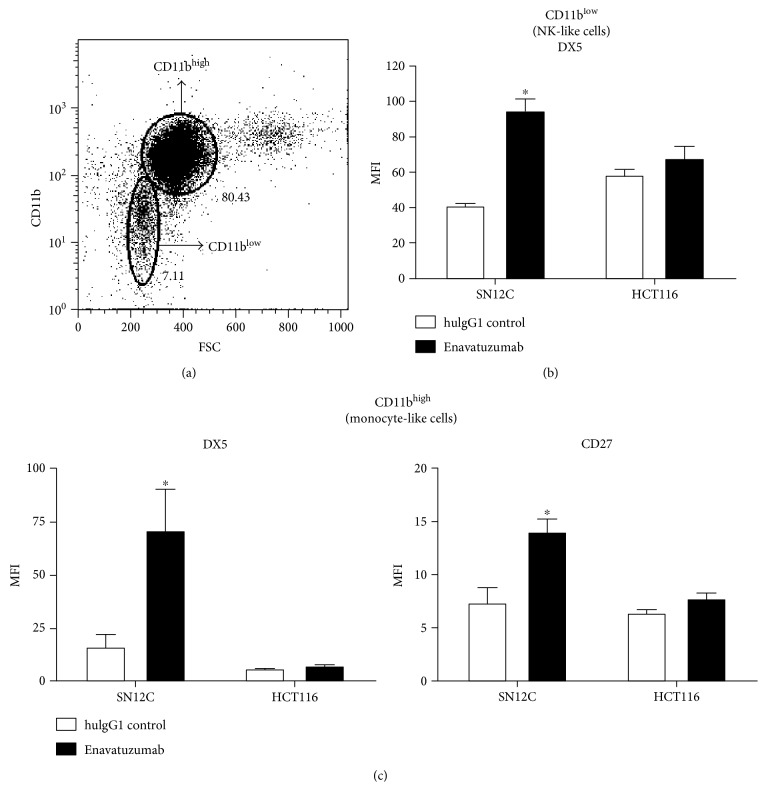
Enavatuzumab activated immune effector cells in responding tumor xenograft model. Splenocytes were isolated from tumor-bearing mice after enavatuzumab treatment and phenotyped by flow cytometry. (a) CD45^+^ cells were gated out from dead cells and other cell types. Of the live CD45^+^ cells, monocyte (CD11b^high^) and NK-like (CD11b^low^) cells were gated based on cell size and CD11b expression. (b, c) Splenocytes from SN12C or HCT116 tumor-bearing mice were assessed for DX5 and CD27 on monocyte-like cells (CD45^+^CD11b^high^) and for DX5 on NK-like cells (CD45^+^CD11b^low^) after enavatuzumab treatment. Enavatuzumab treatment significantly upregulated expression of both markers in SN12C tumor-bearing mice (*n* = 6, ^∗^*p* < 0.05).

The finding that not all xenograft tumors tested were sensitive to enavatuzumab is not consistent with *in vitro* results showing that enavatuzumab was able to induce ADCC and immune cell activation efficiently on all TweakR-positive tumor cell lines tested. This raised the question of whether enavatuzumab might differentially activate effector cells *in vivo*. To address this, splenocytes isolated from tumor-bearing mice after enavatuzumab treatment were assessed for levels of activation markers. NK-like and monocyte-like cells were gated based on cell size and CD11b expression (Figure 3(a)). The activation markers DX5 and CD27 on monocyte-like cells (CD11b high) and DX5 on NK-like cells (CD11b low) were found to be up-regulated after enavatuzumab treatment in responder xenografts that rely on ADCC, such as SN12C tumor-bearing mice, but not in mice bearing HCT116 tumors which do not respond to treatment *in vivo* (Figures 3(b) and 3(c)).

In SN12C tumor-bearing mice, up-regulation of activation markers after enavatuzumab treatment was observed on splenocytes, indicating systemic activation of immune cells. As this activation is unlikely to be mediated by enavatuzumab through mouse TweakR on the immune cells, the function of enavatuzumab on mouse immune effector cells is likely through Fc-Fc*γ*R ligation; such “bridging” between the Fc*γ*R on immune cells and TweakR on the tumor target cells, as suggested in the PBMC-tumor cell co-culture studies, is probably required for triggering effector cell activation *in vivo*.

### 3.3. Enavatuzumab Promoted Infiltration and Migration of Effector Cells into Responder Tumor Xenografts as well as toward Tumor Cells Cultured *In Vitro*

**Figure 4:**
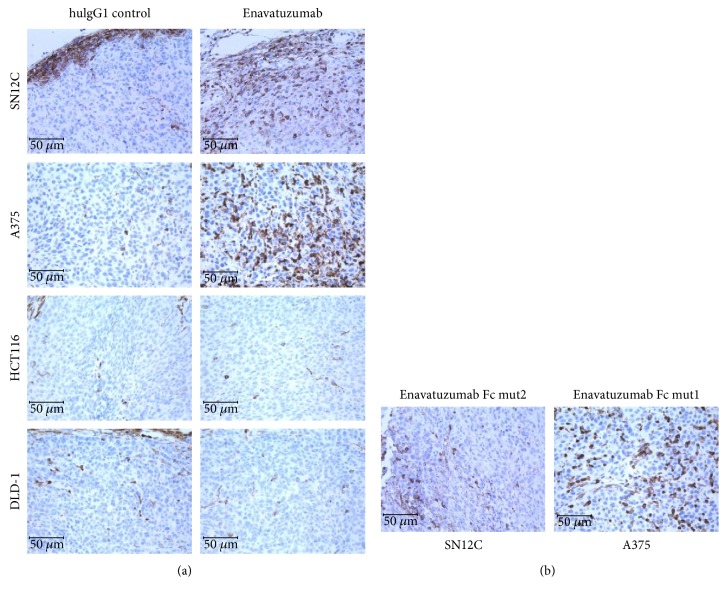
Enavatuzumab treatment results in increased infiltration of CD45^+^ cells into tumors sensitive to antibody treatment. Established xenograft tumors were treated with enavatuzumab or a control antibody (a) or Fc mutants (b) on day 0 and day 2 or 3, and the tumors were harvested on day 4 for immunohistochemical staining for mouse CD45.

**Figure 5:**
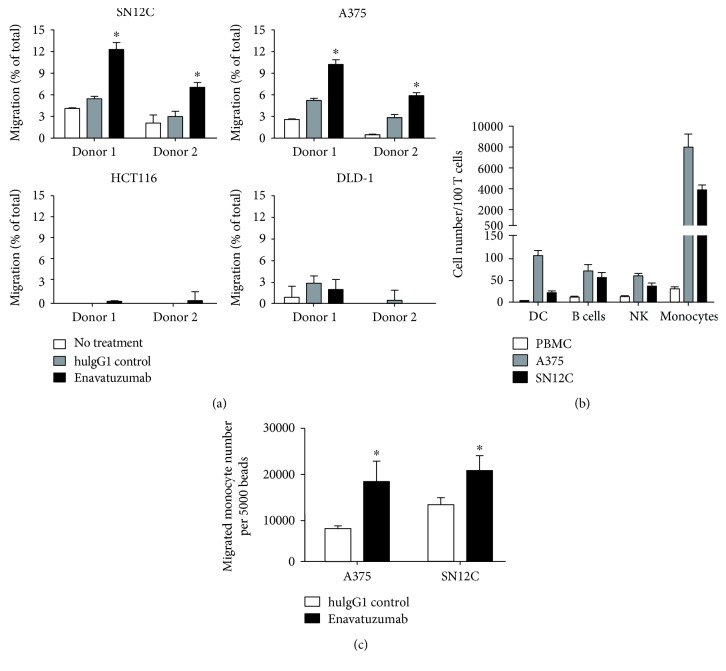
Human effector cells can migrate toward enavatuzumab-treated tumor cells in culture. (a) SN12C, A375, HCT116, and DLD-1 cells were plated into the bottom well of 24-well Transwell plates and treated with enavatuzumab or a control antibody. 24 hr later, PBMCs were added to the top of the Transwell and incubated for an additional 4 hr, after which the number of PBMCs that had migrated to the bottom chamber was quantified by flow cytometry and is represented as a percentage of the total number of PBMCs added. Enavatuzumab significantly increased PBMC migration toward SN12C and A375 cells (∗, *n* = 4, *p* < 0.05) but had no effect on migration toward HCT116 or DLD-1 cells. (b) The phenotype of PBMCs prior to migration was compared to that of immune cells that had migrated toward A375 and SN12C cells after enavatuzumab treatment. The numbers of dendritic cells (CD11c^+^CD3^−^CD20^−^CD56^−^CD16^−^CD14^−^), B cells (CD20^+^), NK cells (CD3^−^CD56/CD16^+^), and monocytes (CD14^+^) were quantified and are expressed relative to the number of T cells in each population. (c) The absolute number of monocytes that migrated toward A375 and SN12C cells after antibody treatment was quantified and is expressed as the number per 5000 counting beads (∗, *n* = 4, *p* < 0.05).

To better understand the interaction between tumor cells and immune cells mediated by enavatuzumab *in vivo*, we assessed the xenograft tumors for the presence of immune cells by immunohistochemistry. While few immune cells were observed within the tumors after treatment with a control antibody, enavatuzumab treatment stimulated marked immune cell infiltration into SN12C and A375 tumors, both of which were sensitive to enavatuzumab (Figure 4(a)). The infiltration of immune cells into these tumors was not dependent on ADCC capability, as enavatuzumab and Fc mutant variants stimulated immune cell infiltration to similar extents (Figure 4(b)). In contrast, enavatuzumab did not stimulate infiltration of immune cells into HCT116 or DLD-1 tumors (Figure 4(a)), both of which were resistant to enavatuzumab *in vivo*. These results suggested that enavatuzumab treatment of sensitive tumors, but not resistant tumors, stimulated the migration of immune cells into the tumor. To simulate the effector cell infiltration observed in animal models, we used a Transwell assay to assess the ability of enavatuzumab to stimulate the migration of human immune cells toward tumor cells. Enavatuzumab treatment of both SN12C and A375 cells resulted in significantly increased migration of immune effector cells toward the tumor cells (Figure 5(a)). In contrast, treatment of HCT116 or DLD-1 cells with enavatuzumab did not stimulate immune cell migration. Phenotyping the immune cells that had migrated toward SN12C or A375 cells showed that monocytes were the predominant migrating immune cell type, as seen by the significant increase in this population relative to the starting PBMCs (Figure 5(b)). NK cells, dendritic cells (DC), and B cells were also enriched in the migrated population. However, only monocyte migration was significantly increased by enavatuzumab treatment of tumor cells (Figure 5(c)).

### 3.4. Chemokines Released from Enavatuzumab-Treated Tumor Cells Were Critical for Effector Cell Migration

**Figure 6:**
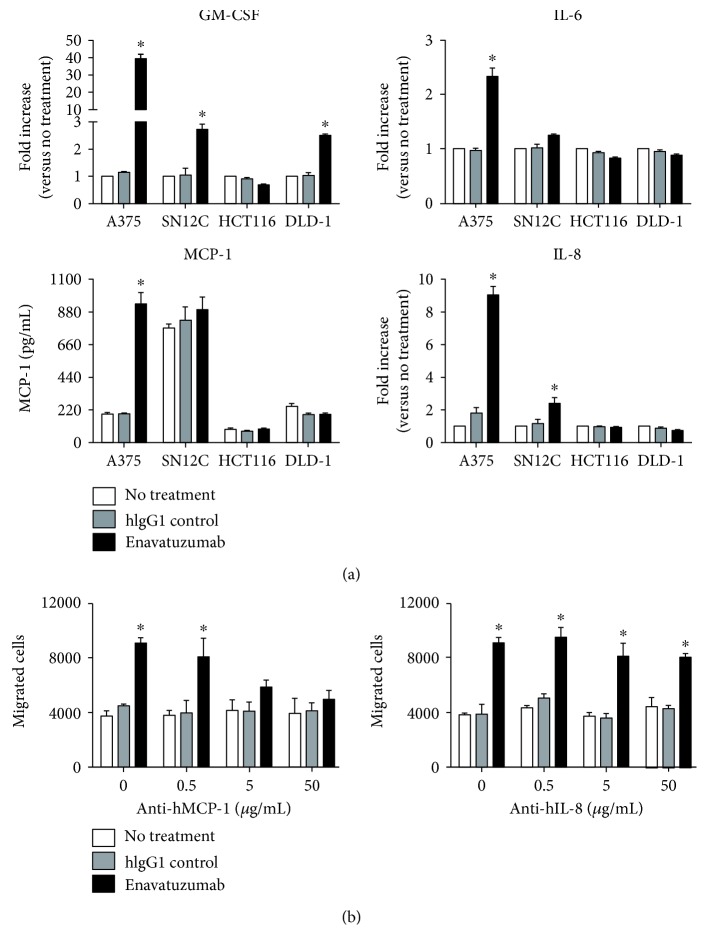
Enavatuzumab stimulates an increase in cytokine levels in some tumor cell cultures. (a) A375, SN12C, HCT116, and DLD-1 cells were treated with enavatuzumab or a control antibody for 24 hr, after which the levels GM-CSF, IL-6, IL-8, and MCP-1 were measured in the supernatants by Luminex multiplex assays (*n* = 4, ^∗^*p* < 0.05). (b) A375 cells were plated into the bottom of Transwell plates and treated with enavatuzumab or a control antibody for 24 hr, after which antibodies blocking MCP-1 (left) or IL-8 (right) were added for 0.5 hr. PBMCs were then added to the top of the Transwell; 4 hr later, the number of immune cells that had migrated toward the tumor cells was quantified by flow cytometry (*n* = 4, ^∗^*p* < 0.05).

The observed ability of enavatuzumab to stimulate migration of immune cells is likely mediated by cytokines released from the tumor cells. TWEAK has been shown to stimulate the release of cytokines and chemokines from a number of cell types [4, 5]; as a TweakR agonist, enavatuzumab would also be expected to have this function. Indeed, enavatuzumab stimulated release of multiple chemokines from A375 cells, including GM-CSF, IL-8, IL-6, and MCP-1 (Figure 6(a)). Treatment of SN12C with enavatuzumab had a more limited effect, resulting in increased GM-CSF and IL-8 levels, although untreated SN12C endogenously expressed high levels of MCP-1. Enavatuzumab had a much reduced effect on cytokine release by HCT116 or DLD-1, with only an increase in GM-CSF released by DLD-1 in response to enavatuzumab (Figure 6(a)).

To test the hypothesis that chemokines mediated enavatuzumab-induced migration of immune cells toward tumor cells, we tested the ability of enavatuzumab to stimulate immune cell migration in the presence of chemokine-blocking antibodies. An IL-8-blocking antibody had no effect on the migration of immune cells toward A375 cells; however, an anti-MCP-1 antibody prevented enavatuzumab-stimulated immune cell migration in a dose-dependent manner (Figure 6(b)), with a marked reduction in monocyte population (Supplemental Figure 1 available online at https://doi.org/10.1155/2017/5737159).

**Figure 7:**
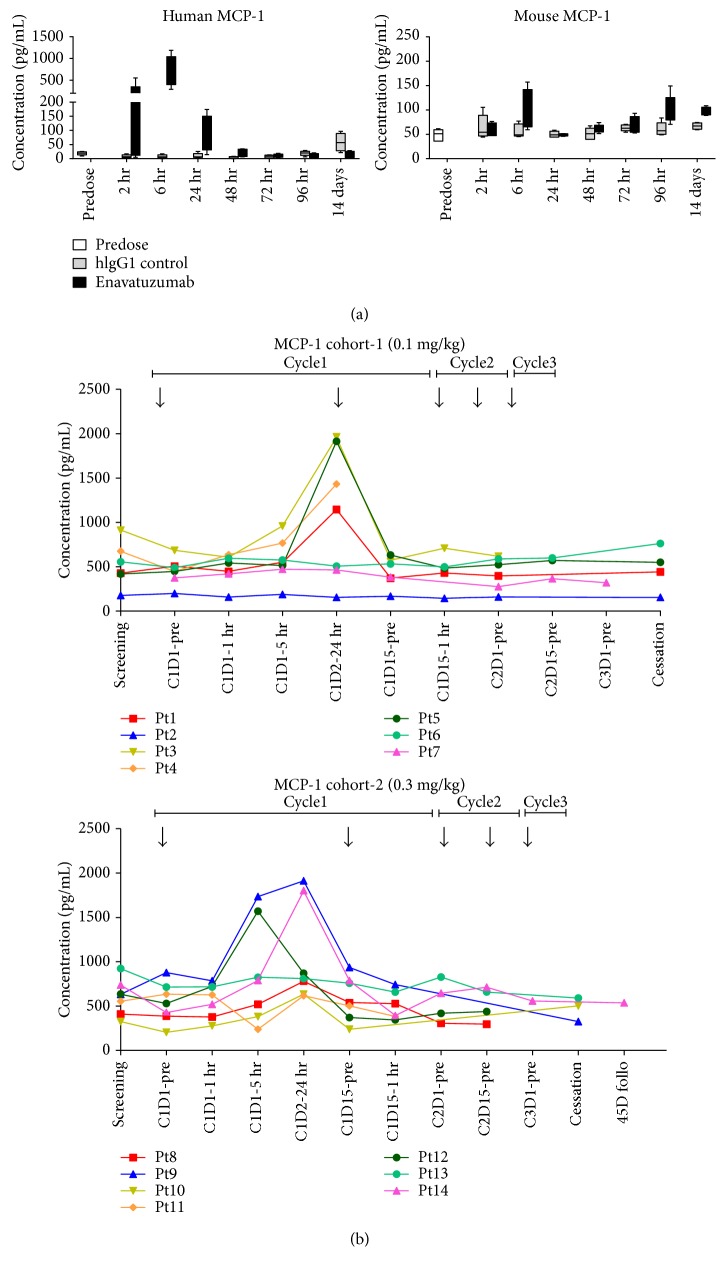
Enavatuzumab treatment stimulates MCP-1 secretion in mouse xenograft models and in human cancer patients. (a) Mice bearing established A375 tumors were given a single dose of enavatuzumab or a control antibody. Human MCP-1 (left) and mouse MCP-1 (right) were measured by Luminex in the serum of 5 mice in each dosing group at the indicated times after treatment. Data from 5 mice are shown as median and interquartile range at each time point in the graph. (b) Patients were treated with enavatuzumab at 0.1 mg/kg (top) or 0.3 mg/kg (bottom) every two weeks, with dosing days indicated by the arrows above each graph. MCP-1 was measured in serum samples by Luminex. Each colored line represents data from one patient and patient (Pt) numbers are also listed.

Having shown that enavatuzumab treatment of tumor cells stimulated release of multiple cytokines and chemokines *in vitro* and that MCP-1 appeared to be a key functional chemokine in immune cell migration, we next tested whether enavatuzumab stimulated release of MCP-1 *in vivo*. A375 tumor-bearing mice were administered a single dose of enavatuzumab, after which serum from mice was assessed for human or mouse MCP-1 levels. Enavatuzumab treatment resulted in a marked increase in circulating human MCP-1, which peaked 6 hrs after antibody injection (Figure 7(a)). Enavatuzumab stimulated a modest elevation in mouse MCP-1 at a single time point, 6 hrs after treatment. Treatment of mice bearing SN12C tumors with enavatuzumab also resulted in elevated circulating human and mouse MCP-1 levels (Supplemental Figure 2).

To translate the preclinical finding that enavatuzumab stimulated cytokine release from tumor cells *in vitro* and *in vivo* as a mechanism to mediate the migration of immune effector cells into tumors, we next assessed whether enavatuzumab might also have an effect on circulating chemokine levels in cancer patients. MCP-1 was measured in the serum from phase 1 subjects at various times after enavatuzumab treatment. At all dose levels tested (0.1–1.5 mg/kg), postdose elevations in MCP-1 were observed, with 13/30 patients exhibiting at least a 2-fold increase over baseline at 5 hr and/or 24 hr after the first infusion (Figure 7(b)). The increased level of MCP-1 could be of value as a potential biomarker for enavatuzumab biological activity in patients.

## 4. Discussion

Enavatuzumab is a functional anti-TweakR human IgG1 antibody that inhibits tumor cell growth through direct signaling and also kills tumor cells through ADCC. The current study confirmed that enavatuzumab can induce cytotoxicity of all TweakR-positive tumor cells tested by ADCC *in vitro*. However, not all TweakR-positive cells which can be lysed by ADCC *in vitro* were sensitive to enavatuzumab treatment in mouse xenograft tumor models. We hypothesize that tumor cells sensitive to enavatuzumab treatment *in vivo* actively recruit immune effector cells and the presence of immune effector cells within the tumor is critical for antibody-mediated tumor cell killing.

Immunohistochemistry (IHC) staining demonstrated strong presence of CD45^+^ leukocytes within xenograft tumors sensitive to enavatuzumab treatment, and very few CD45^+^ cells were found in nonresponding tumors. The infiltration of leukocytes into sensitive tumors was rapid and occurred within 3 days after the first dose; thus, it is unlikely that the leukocytes were recruited as a result of tumor necrosis at later time points (Supplemental Figure 3). In the *in vitro* Transwell assay, enavatuzumab stimulated the responder tumor cell lines to produce a number of cytokines and chemokines which attracted human leukocytes. In contrast, nonresponding lines produced few chemokines after exposure to enavatuzumab. In experiments using conditioned media, MCP-1 was found to be the major chemokine responsible for leukocyte infiltration into tumors. These data suggest that stimulation of TweakR signaling by enavatuzumab not only leads to tumor growth inhibition as previously reported [2] but can also result in chemokine release and leukocyte infiltration. The ability to stimulate leukocyte infiltration into tumors may explain the potent antitumor activity of enavatuzumab in responding tumor models. In xenograft models conducted in immune-deficient mice, the major leukocytes are neutrophils, monocytes, and NK-like cells, all of which express Fc*γ*R [25]. The presence of Fc*γ*R-positive leukocytes within the tumor would allow the antibody to bridge leukocytes and tumor targets through binding to Fc*γ*R and TweakR, respectively, thus simultaneously modulating the functions of both tumor target cells and leukocytes.

Fc*γ*R binding has been demonstrated to drive direct signaling in target cells for antibody targeting members of TNFRSF, such as anti-DR5 [21]. It is well-known that members of the TNFRSF need to be oligomerized to initiate downstream signaling. Antibodies clustered by Fc*γ*R expressed on neighboring cells likely facilitate target receptor oligomerization and subsequent downstream signaling. A recent study on anti-TweakR antibodies confirmed this concept and provided evidence that antibodies can enhance TweakR-mediated signaling through Fc*γ*R binding [26]. One of the biological responses resulting from TweakR direct signaling is cytokine or chemokine production [4, 5]. Crosslinking provided by leukocytes can enhance TweakR signaling; thus, the subsequent cytokine or chemokine production from tumor cells could further increase leukocyte recruitment. This positive feedback loop created by leukocyte infiltration can therefore amplify the biological activity of enavatuzumab. The other biological response from TweakR direct signaling is tumor growth inhibition or cell death [7–9]. We have shown that crosslinking the Fc region of enavatuzumab using a soluble secondary antibody or by immobilizing enavatuzumab enhanced growth inhibition in responding tumor cell lines *in vitro* [10, 19]. It is possible that tumor-infiltrating leukocytes provide similar crosslinking through Fc-Fc*γ*R interactions and therefore enhance the antitumor activity of enavatuzumab *in vivo*. This antitumor activity of enavatuzumab is independent of its ADCC function. Such activity is likely a major mechanism driving the antitumor activity in the A375 model, where enavatuzumab and an ADCC-null Fc mutant (hIgG1-LALA) of enavatuzumab exhibited equivalent antitumor activity. This Fc mutant retained some mouse Fc*γ*R binding which might be sufficient to provide the crosslinking required to promote TweakR signaling, leading to tumor growth inhibition.

By bridging leukocytes and tumor cells, enavatuzumab can not only stimulate TweakR signaling on tumor cells but also enhance the activation of Fc*γ*R-bearing leukocytes. Human IgG1 antibodies' binding to activating Fc*γ*R and engagement of activating Fc*γ*R by antibody-antigen complexes may lead to leukocyte activation, cytokine release, and ADCC stimulation [20, 27]. Consistent with this notion, enavatuzumab activated NK cells and monocytes by modulating expression of cell surface markers when human PBMCs were cocultured with tumor cells. Since neither NK cells nor monocytes express TweakR [2, 24], activation of these cells is mediated by Fc-Fc*γ*R interactions and only when PBMCs were cocultured with TweakR-expressing tumor cells. In the absence of antigen engagement on the tumor cells, enavatuzumab treatment did not alter the expression of activation markers on PBMCs. These *in vitro* data suggested that crosslinking through tumor target binding was required for Fc*γ*R-mediated effector cell activation and is analogous to the ability of crosslinking through Fc*γ*R engagement to promote signaling through TweakR as discussed above. Enavatuzumab also activated leukocytes *in vivo*, as evidenced by the upregulation of activation markers expressed on the leukocytes from the spleens of mice bearing xenograft tumors sensitive to enavatuzumab. Given that the sensitive tumors were capable of attracting leukocytes after treatment, leukocytes might be activated while circulating through tumor xenografts in the presence of the antibody. Further evidence of *in vivo* effector cell activation by enavatuzumab comes from the observation that the production of mouse cytokines was enhanced by enavatuzumab in sensitive tumor models (Figure 7(a), Supplemental Figure 2). Since enavatuzumab does not bind to mouse TweakR, the mouse cytokines were likely secreted from host leukocytes activated by antibody Fc*γ*R engagement. Assessing cell surface marker expression and cytokine production associated with *in vivo* effector cell activation provided feasible ways to monitor biomarkers reflecting the biological activity of enavatuzumab.

Many studies have suggested that the activation of effector cells is sufficient to induce target cell killing via ADCC [28, 29]. In this study, however, we showed that tumor cell killing also required a sufficient quantity of activated effector cells, since optimal cell cytotoxicity mediated by enavatuzumab could only be achieved at higher E : T ratios than that was required for optimal activation of effector cells in culture. Consistent with this finding, a previous study assessing the ADCC capacity of effector cells derived from trastuzumab-treated patients showed that response to trastuzumab correlated with the numbers of CD56^+^ or CD16^+^ lymphocytes in PBMCs and with the ability of the PBMCs to lyse target cells [30]. Thus, the quantity of effector cells is as important as their functionality in mediating ADCC, suggesting that the active recruitment of leukocytes into tumors may further enhance the antitumor activity of enavatuzumab via ADCC.

Taken together, these data suggest a model to describe the various mechanisms by which enavatuzumab exerts its antitumor activity in xenograft models. Binding of enavatuzumab to its target on tumor cells such as SN12C or A375 initiates signaling through TweakR which leads to cytokine and chemokine production. The tumor-derived cytokines and chemokines then trigger a cascade of biological responses, including leukocyte infiltration, enhanced leukocyte activation by antibody-tumor target engagement, and enhanced tumor signaling through antibody crosslinking via effector cells. The increased number of activated effector cells within the tumor facilitates tumor cell killing through ADCC, while the enhanced tumor cell signaling through TweakR further promotes direct tumor growth inhibition.

MCP-1 was identified as a chemokine likely responsible for enavatuzumab-stimulated leukocyte infiltration in xenograft models. MCP-1 is known to attract monocytes and has previously been shown to be secreted by tumor cells in response to TweakR stimulation [11, 31]. It has also been reported that monocytes and macrophages at the tumor site may contribute to tumor growth inhibition through the release of soluble TWEAK [11, 32]. The results described in this manuscript demonstrate recruitment of monocytes and NK cells toward tumor cells upon MCP-1 release after enavatuzumab treatment, resulting in activation of these innate effector cells and subsequent ADCC or ADCP on tumor cells. Immune-deficient mice carrying xenograft tumors provide a relevant model to test enavatuzumab in contact with innate immune cells. In an immune-competent environment, there are studies showed that macrophage plays an important role in depleting target cells [33, 34] as well as in mediating antitumor immunity [35]. However, other studies showed that chronic activation of macrophage recruited into tumor may suppress adaptive antitumor immunity in syngeneic setting [36, 37]. The precise role of macrophages in the tumor microenvironment depends on multiple factors, including the phenotype of the cells at a given time, the timing of treatment, and the tumor model in which the studies are performed [38]. To study the effects of acute recruitment and activation of monocytes/macrophages by functional anti-TweakR mAb on tumor cells, surrogate murine antibody will be required for testing in mouse-syngeneic tumor models, which could provide a better translation to understand the role of MCP-1 induced by enavatuzumab in cancer patients. Nevertheless, the rapid induction of MCP-1 by enavatuzumab in clinical studies suggests the potential for this molecule to be a biomarker of biological activity for enavatuzumab. Monitoring this marker may provide a better understanding of the relationship of pharmacokinetics, pharmacodynamics and toxicity profile of enavatuzumab, which might facilitate the development of an appropriate dosing regimen in clinical studies.

## Supplementary Material

The information of supplementary materials are as follows: Supp Figure 1. A375 cells were plated into the bottom of Transwell® plates and treated with enavatuzumabor a control antibody for 24 hr, after which antibody blocking MCP-1 was added for 0.5 hr. PBMCs were then added to the top of the Transwell®; 4 hrlater, the number of immune cells that had migrated toward the tumor cells was quantified by flow cytometry. Supp Figure 2. Mice bearing established SN12C tumors were given a single dose of enavatuzumab or a control antibody at 3 mg/kg. At the indicated times, blood was collected and the levels of human MCP-1 (upper) and mouse MCP-1 (lower) were measured in the serum by Luminex®. Supp Figure 3. SN12C tumor-bearing mice were dosed with enavatuzumab, an Fc mutant variant of enavatuzumab, or a control antibody on day 0, 3, 5, 7, 9, 11, and 13. On the indicated days, tumors were harvested from 3 animals in each dosing group, and stained for mouse CD45 by immunohistochemistry. One representative image is depicted.
