# Supplementary material for: Enavatuzumab, a Humanized Anti-TWEAK Receptor Monoclonal Antibody, Exerts Antitumor Activity through Attracting and Activating Innate Immune Effector Cells
Source: J Immunol Res. 2017 Sep 17;2017:5737159. doi: 10.1155/2017/5737159 (PMC5623805; doi:10.1155/2017/5737159)
Supplement: Supplementary file 1 — The information of supplementary materials are as follows: Supp Figure 1. A375 cells were plated into the bottom of Transwell® plates and treated with enavatuzumabor a control antibody for 24 hr, after which antibody blocking MCP-1 was added for 0.5 hr. PBMCs were then added to the top of the Transwell®; 4 hrlater, the number of immune cells that had migrated toward the tumor cells was quantified by flow cytometry. Supp Figure 2. Mice bearing established SN12C tumors were given a single dose of enavatuzumab or a control antibody at 3 mg/kg. At the indicated times, blood was collected and the levels of human MCP-1 (upper) and mouse MCP-1 (lower) were measured in the serum by Luminex®. Supp Figure 3. SN12C tumor-bearing mice were dosed with enavatuzumab, an Fc mutant variant of enavatuzumab, or a control antibody on day 0, 3, 5, 7, 9, 11, and 13. On the indicated days, tumors were harvested from 3 animals in each dosing group, and stained for mouse CD45 by immunohistochemistry. One representative image is depicted. [file 5737159.f1.pdf]

Supplemental Figures

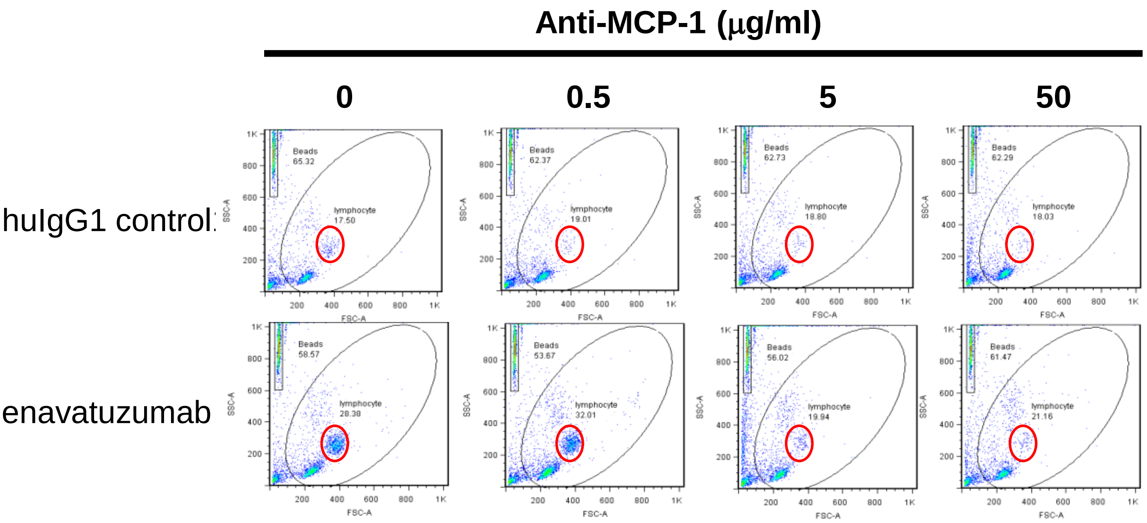

Supp Figure 1. A375 cells were plated into the bottom of Transwell® plates and treated with enavatuzumab or a control antibody for 24 hr, after which antibody blocking MCP-1 was added for 0.5 hr. PBMCs were then added to the top of the Transwell®; 4 hr later, the number of immune cells that had migrated toward the tumor cells was quantified by flow cytometry.

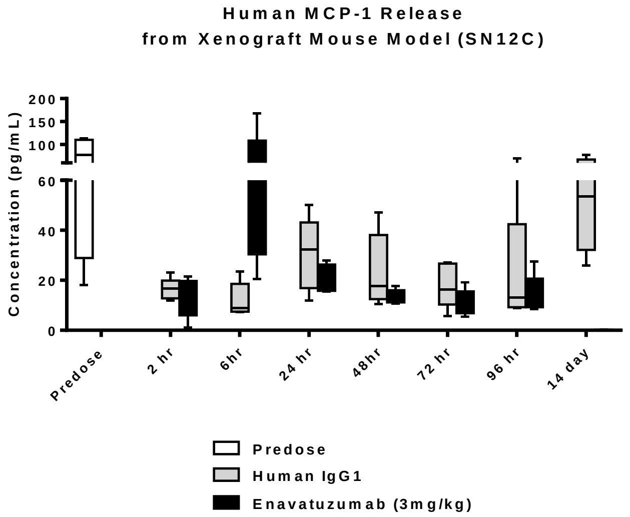

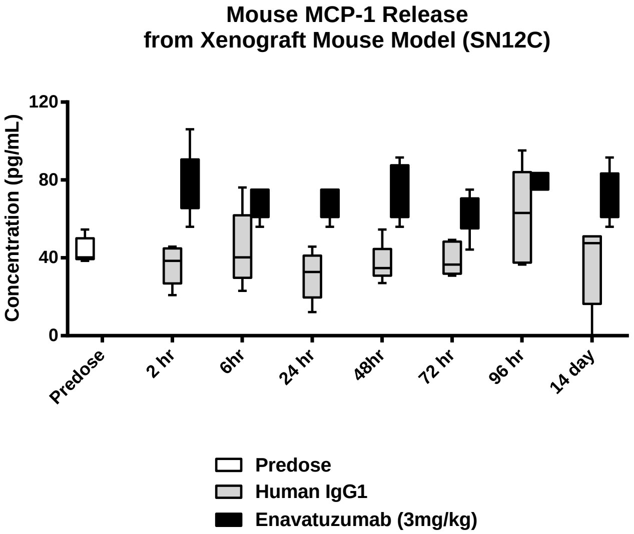

Supp Figure 2. Mice bearing established SN12C tumors were given a single dose of enavatuzumab or a control antibody at 3 mg/kg. At the indicated times, blood was collected and the levels of human MCP-1 (upper) and mouse MCP-1 (lower) were measured in the serum by Luminex®.

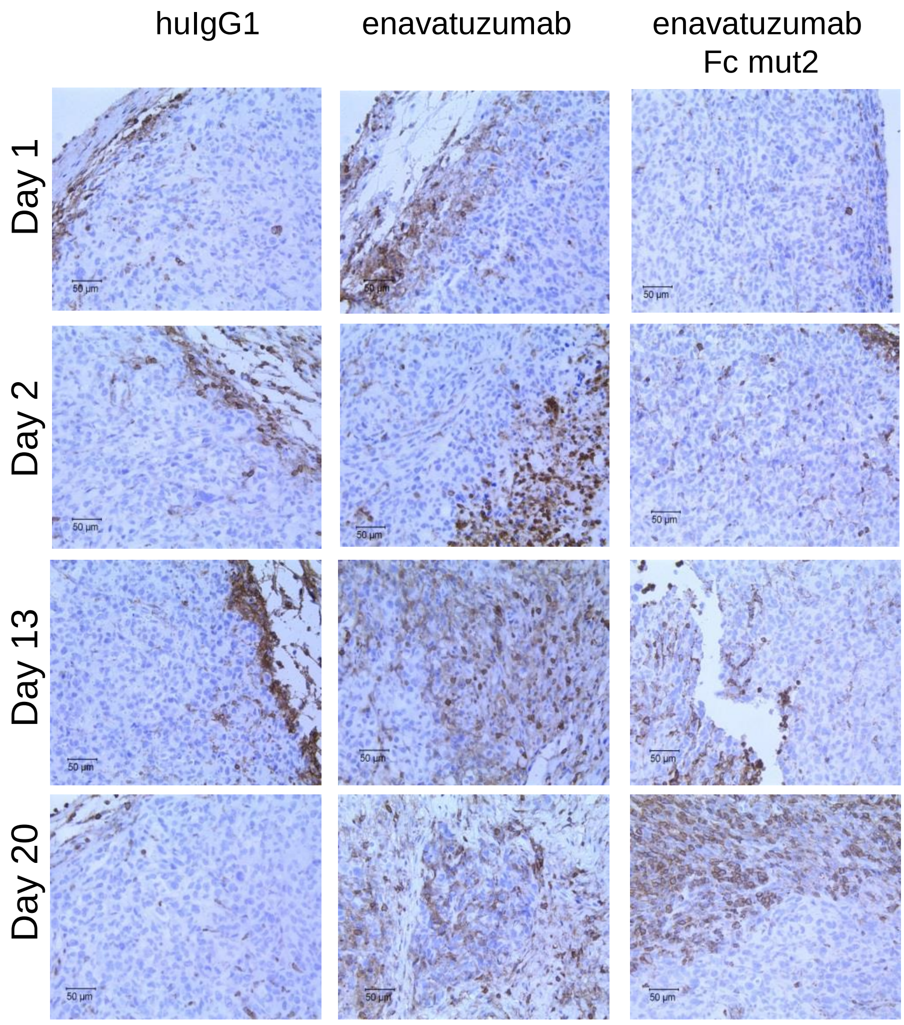

Supp Figure 3. SN12C tumor-bearing mice were dosed with enavatuzumab, an Fc mutant variant of enavatuzumab, or a control antibody on day 0, 3, 5, 7, 9, 11, and 13. On the indicated days, tumors were harvested from 3 animals in each dosing group, and stained for mouse CD45 by immunohistochemistry. One representative image is depicted.
